# A nonsense mutation in the tyrosinase gene causes albinism in water buffalo

**DOI:** 10.1186/1471-2156-13-62

**Published:** 2012-07-20

**Authors:** Maria Cecília Florisbal Damé, Gildenor Medeiros Xavier, José Paes Oliveira-Filho, Alexandre Secorun Borges, Henrique Nunes Oliveira, Franklin Riet-Correa, Ana Lucia Schild

**Affiliations:** 1 Brazilian Agricultural Research Corporation, Embrapa, Pelotas, Rio Grande do Sul, 96.001-970, Brazil; 2 Veterinary Hospital, Federal University of Campina Grande, Patos, Paraíba, 58700-000, Brazil; 3 Laboratory of Molecular Biology of Department of Veterinary Clinical Science / College of Veterinary Medicine and Animal Science, Univ Estadual Paulista (Unesp), Botucatu, Sao Paulo, 18618-970, Brazil; 4 Laboratory of Molecular Biology of Department of Veterinary Clinical Science / College of Veterinary Medicine and Animal Science, Univ Estadual Paulista (Unesp), Botucatu, Sao Paulo, 18618-970, Brazil; 5 Departamento de Zootecnia / Faculdade de Ciências Agrárias e Veterinárias, Univ Estadual Paulista (Unesp), Jaboticabal, Sao Paulo, 14884-900, Brazil; 6 Veterinary Hospital, Federal University of Campina Grande, Patos, Paraíba, 58700-000, Brazil; 7 Veterinary Diagnostic Laboratory, Federal University of Pelotas, Pelotas, Rio Grande do Sul, 96010-900, Brazil

**Keywords:** Albinism, Buffalo, Nonsense mutation, Stop codon, Tyrosinase

## Abstract

**Background:**

Oculocutaneous albinism (OCA) is an autosomal recessive hereditary pigmentation disorder affecting humans and several other animal species. Oculocutaneous albinism was studied in a herd of Murrah buffalo to determine the clinical presentation and genetic basis of albinism in this species.

**Results:**

Clinical examinations and pedigree analysis were performed in an affected herd, and wild-type and OCA tyrosinase mRNA sequences were obtained. The main clinical findings were photophobia and a lack of pigmentation of the hair, skin, horns, hooves, mucosa, and iris. The results of segregation analysis suggest that this disease is acquired through recessive inheritance. In the OCA buffalo, a single-base substitution was detected at nucleotide 1,431 (G to A), which leads to the conversion of tryptophan into a stop codon at residue 477.

**Conclusion:**

This premature stop codon produces an inactive protein, which is responsible for the OCA buffalo phenotype. These findings will be useful for future studies of albinism in buffalo and as a possible model to study diseases caused by a premature stop codon.

## Background

Oculocutaneous albinism (OCA) is an autosomal recessive hereditary disorder characterized by a partial or total absence of melanin in the hair, skin and eyes in humans and animals. Tyrosinase catalyzes the first two steps of melanin biosynthesis, i.e., the hydroxylation of tyrosine to 3, 4-dihydroxyphenylalanine (DOPA) and the subsequent oxidation of DOPA to dopaquinone [1]. Loss of tyrosinase (TYR) mRNA expression prevents melanin synthesis, thereby causing albinism [2].

Cases of OCA caused by mutations in the TYR gene that encodes tyrosinase have been documented in several mammalian species, including humans [2-8]. Mutations in other genes (TYRP1, OCA2) have also been associated with different types of OCA [9-11] and ocular albinism (OA1) in humans [12]. Mutations in the human LYST and HPS genes are responsible for OCA and are associated with Chediak-Higashi [13] and Hermansky-Pudlak [14] syndromes, respectively. Mutation of the LYST gene in cattle is also responsible for OCA and for a syndrome similar to the human form of Chediak-Higashi [15].

Oculocutaneous albinism has previously been reported in an Anatolian buffalo calf in Turkey [16] and in a herd of Murrah buffalo in Brazil [17]. Additionally, a white water buffalo calf was described in 1925 [18] in which every hair on the body was white but the eyes exhibited normal pigmentation. However, the mutation responsible for these cases was not investigated. The objective of the present study is to report the clinical and genetic features of albinism in a herd of water buffalo of the Murrah breed. We report the sequence of the TYR gene in both wild-type and OCA Murrah buffalo and describe a nonsense mutation that produces a premature stop codon that results in an abbreviated protein product. This abnormal protein is responsible for OCA in these animals.

## Methods

### Epidemiology and clinical signs

The disease was studied in a herd of Murrah buffalo in the state of Rio Grande do Sul in southern Brazil (S 31°49’03” and W 52°26’25”). The data were obtained by reviewing the genetic records of the experimental herd (1297 animals born into the herd beginning in 1996), and the affected buffalo were subjected to clinical examination. The active sires in 1996 were considered as a base population to the estimate herd's consanguinity. However, the complete genealogical data from these sires and from those sires incorporated into the herd after 1996 were used for segregation analysis. The segregation analysis and the estimation of allele frequencies were performed using Geneprob software (Gene Probe Inc., Atlanta, GA) [19]. The coefficient of consanguinity was calculated using in-house-developed software based on the algorithm described by Meuwissen and Luo [20]. Under a hypothesis of Mendelian recessive inheritance with complete penetrance, the frequency of albinism among offspring that were from sires considered to be unequivocally heterozygous and were mated either with their own daughters or with daughters from sires sharing the same characteristics was tested to determine if the observed frequency of albinism agreed with the expected frequency, considering a binomial distribution (n, p=1/8).

### Animals

All experiments were performed according to the regulations of the Sao Paulo State University Institutional Animal Care and Use Committee (231/2011-CEUA). Skin samples were collected from six Murrah buffalo with a clinical diagnosis of OCA (III-110, VII-690, X-253, X-257, X-273, and XII-10a) and from four normal Murrah buffalo with black skin (control group). The TYR mRNA sequence was then determined for each subject. The animals used as the control group belonged to a herd from Sao Paulo State (S 22°88’78” and W 48°50’07”). Blood samples for single nucleotide polymorphism (SNP) DNA sequencing were collected from 15 Murrah buffalo, including nine albinos (III-110, VII-690, IX-167, X-253, X-257, X-273, XI-290, XI-351, and XII-10a), four heterozygotes (VI-523, VI-639, VIII-7, and VIII-553) and two wild-type buffalo (control herd).

### Tyrosinase mRNA sequencing in the normal buffalo

Skin biopsies were obtained from four black wild-type buffalo using a 5-mm punch (Kolplast, Sao Paulo, Brazil). The samples were frozen in liquid nitrogen immediately after the biopsy procedure and then stored at -80°C until RNA purification. Total RNA was isolated from the skin samples using the RNeasy^®^ Mini Kit (Qiagen^®^, Valencia, CA) according to the manufacturer’s instructions. The relative purity and quality of the isolated RNA were determined with a Nanodrop^®^ 2000 Spectrophotometer (Thermo Scientific^TM^, Wilmington, DE). The absorbance ratio (A260/A280 nm) exceeded 1.8 for all samples. To ensure the complete removal of all genomic DNA, 2 μg of total RNA was incubated with RQ1 RNase-Free DNase (Promega, Madison, WI) according to the manufacturer’s instructions. First-strand cDNA synthesis was performed using 600 ng of total RNA per 60 ml reaction volume using random hexamers and the ImProm-II^TM^ Reverse Transcription System (Promega) according to the manufacturer’s instructions.

Because the buffalo TYR mRNA sequence is not publicly available, 10 sets of heterologous primers were designed (Table 1) based on the *Bos taurus* TYR mRNA sequence deposited in GenBank^TM^ (NM_181001.2). PrimerQuest^®^ software (Integrated DNA Technologies Inc., Coralville, IA) was used to obtain the TYR mRNA sequence of the normal buffalo.

**Table 1:** **Primers used for RT-PCR amplification of*B. bubalis*tyrosinase mRNA**

	**Name**	**Primer Sequence (5´- 3´)**	**Product Size (bp)**	**Annealing Temperature**
**1**	**ASB.TYR - F1**	TGAAAGGGAAGAGTGTGGCTCCAT	411	60 °C
	**ASB.TYR - R8**	TCGCCTTTCTGTGCAGCGGG		
**2**	**JP.TYR - F2**	AGTCTTGGCCCTCCATCTTT	350	60 °C
	**JP.TYR - R2**	GACTTCAGAGTCCCCAAGCA		
**3**	**ASB.TYR - F6**	TCCCCACGGGCACCTATGGC	419	60 °C
	**ASB.TYR - R1**	GTTGCATAAAGCCTGGCGACTGTT		
**4**	**ASB.TYR - F7**	CATGGGAGGGCGCAACCCTG	533	60 °C
	**ASB.TYR - R2**	AAGGAACCATGTAGGATTCCCGGT		
**5**	**ASB.TYR - F2**	AACAGTCGCCAGGCTTTATGCAAC	439	60 °C
	**ASB.TYR - R2**	AAGGAACCATGTAGGATTCCCGGT		
**6**	**JP.TYR - F4**	GATCTGCCAATGATCCCATC	329	60 °C
	**JP.TYR - R4**	AAGGACAGACCCAACCACAG		
**7**	**ASB.TYR - F3**	TAGAACAAGCACAACGAATCTGGC	377	60 °C
	**ASB.TYR - R3**	AAAGCAAGCACAGGTGGCTTCTAC		
**8**	**ASB.TYR - F4**	ACCGGGAATCCTACATGGTTCCTT	225	60 °C
	**ASB.TYR - R4**	TAAGTCCTCCCAGCACAGCAGTAA		
**9**	**ASB.TYR - F5**	AATGTAGCCCTCCTCCTACTCAGGTA	215	60 °C
	**ASB.TYR - R5**	GGGAACAAGTCATTCCACAATCAAGAGG		
**10**	**JP.TYR - F6**	CAATAGAGCTGGGGCAAAAA	247	60 °C
	**JP.TYR - R6**	TACCAAATGGCATCCTTTCC		

The RT-PCR amplifications were performed in a total volume of 50 μl, which contained 0.3 μM each forward and reverse primer, 5 μl of template cDNA, 25 μl of GoTaq^®^ Green PCR Master Mix (Promega), and 17 μl of nuclease-free water. In addition, a no-template control reaction was performed in duplicate to check for the possible presence of contamination in the preparations. The obtained RT-PCR products were analyzed via 1.5% agarose gel electrophoresis (Invitrogen^TM^, Carlsbad, CA), stained with the Sybr^®^ Safe DNA Gel Stain (Invitrogen^TM^) and then purified using the QIAquick^®^ PCR Purification Kit (Qiagen^®^). The purified products of the correct estimated size were then submitted for direct sequence analysis using BigDye^®^ Terminator v3.1 Cycle Sequencing (Applied Biosystems^TM^, Foster City, CA). Each reaction was performed in quadruplicate using 5 μl of each of the forward and reverse primers and 10 μl of the RT-PCR product. The obtained sequences and electropherograms were analyzed using Sequencing Analysis 5.3.1 software (Applied Biosystems^TM^). The *B. bubalis* TYR mRNA sequence obtained from wild-type buffalo was subjected to BLAST searches (http://blast.ncbi.nlm.nih.gov) against other mammalian TYR sequences deposited in GenBank^TM^ and aligned using CLUSTAL X software.

### Tyrosinase mRNA sequencing of buffalo with OCA

Skin biopsies were performed on the six buffalo with OCA, and RNA purification, reverse transcription, RT-PCR and sequencing were performed as described above to obtain the TYR mRNA sequences. These sequences were then compared to the wild-type *B. bubalis* TYR mRNA sequence using the CLUSTAL X program to identify potential mutations in the TYR mRNA sequence from the buffalo with OCA.

### Single nucleotide polymorphism (SNP) DNA sequencing

Blood sample DNA purification was performed using an Ilustra^TM^ blood genomic Prep Mini Spin Kit (GE Healthcare Life Science, Buckinghamshire, UK) according to the manufacturer’s instructions. PCR for SNP DNA sequencing was performed as described above using only the ASBTYR-F3 and ASBTYR-R3 primers. PCR products of the expected size were purified, sequenced and analyzed as described previously.

## Results

**Figure 1:**
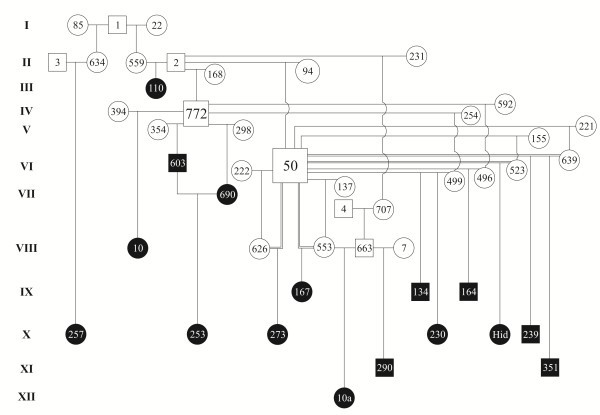
Pedigree of the albino buffalo herd. Black graphics represent the affected individuals.

In the studied herd, some of the cows were initially inseminated with semen imported from Bulgaria (from bull I-1 or II-2). According to the records, despite the fact that these two bulls were from the same Bulgarian stud, they were not related, at least up to two generations, but both grandfathers came from India, indicating a small chance of a previous relationship between them. Unfortunately, we have no information concerning the occurrence of albinism in this Bulgarian stud. During a 12-year period, 1.6% (16/970) of the calves produced were born with albinism. The pedigree of the calves with albinism is presented in Figure 1. With the exception of X-257, which shared kinship with only one of the Bulgarian sires (I-1), all of the albino animals born into the herd shared kinship with both of the imported sires (I-1 and II-2) whose imported semen was used on the farm. The calculated frequency for the recessive allele was 0.0552 and the frequency of albinism in the herd was 16/970. Considering the active sires in 1996 as the founders of the herd, the mean consanguinity coefficient was 6.01% for the 84 animals born in 2009. Among the albino animals, the consanguinity was 14.9%, and only two of the albino animals showed no consanguinity (III-110 and X-257). The results of the segregation analysis showed that the assessed genealogical data agree with the hypothesis of autosomal recessive inheritance. When the numbers of albino offspring from sires considered unequivocally heterozygous mated with their own daughters or with daughters from heterozygous sires were compared using segregation analysis, we obtained a value of 12/73, indicating no significant difference (p>0.11) from the expected value (9.1/73), even ignoring the possibility that the cows could have received the recessive allele from their mothers.

**Figure 2:**
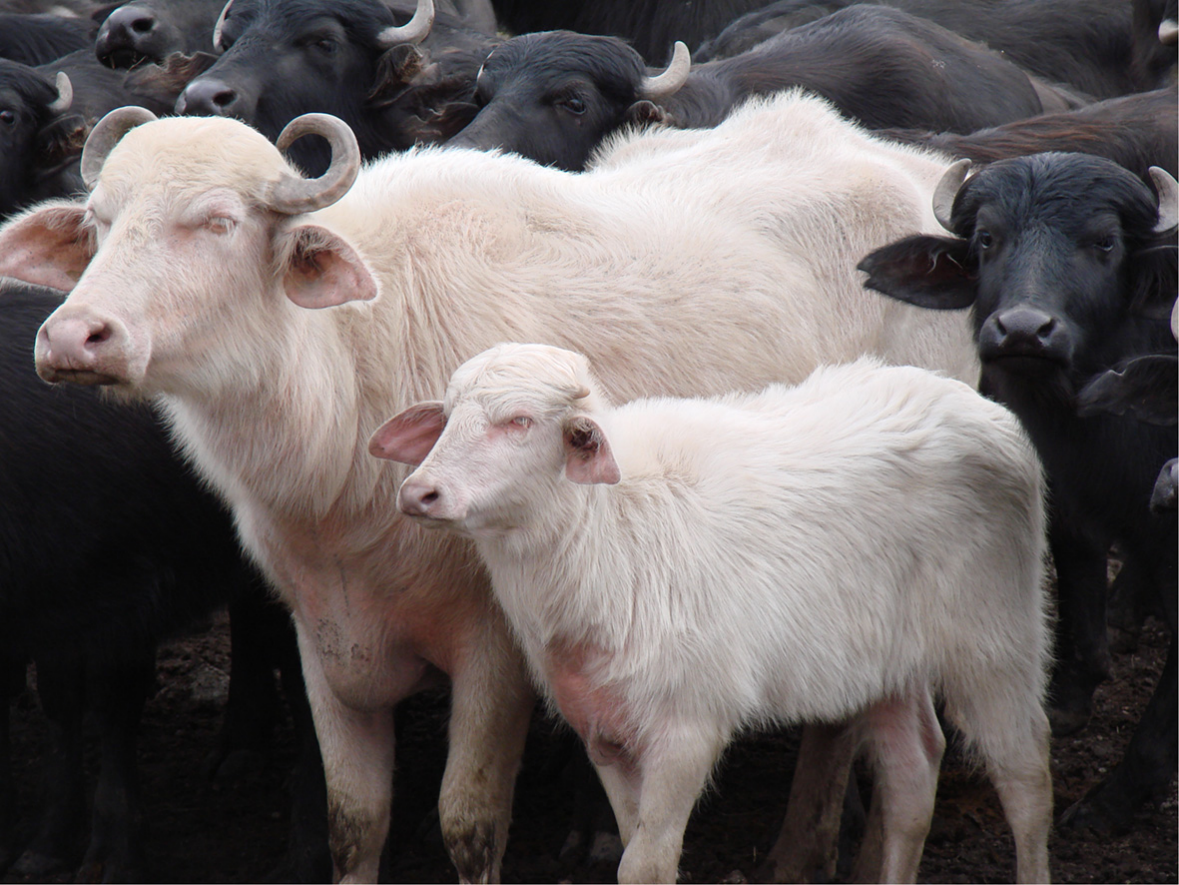
An albino dam and her albino calf (with photophobia) are shown among normal buffalo.

**Figure 3:**
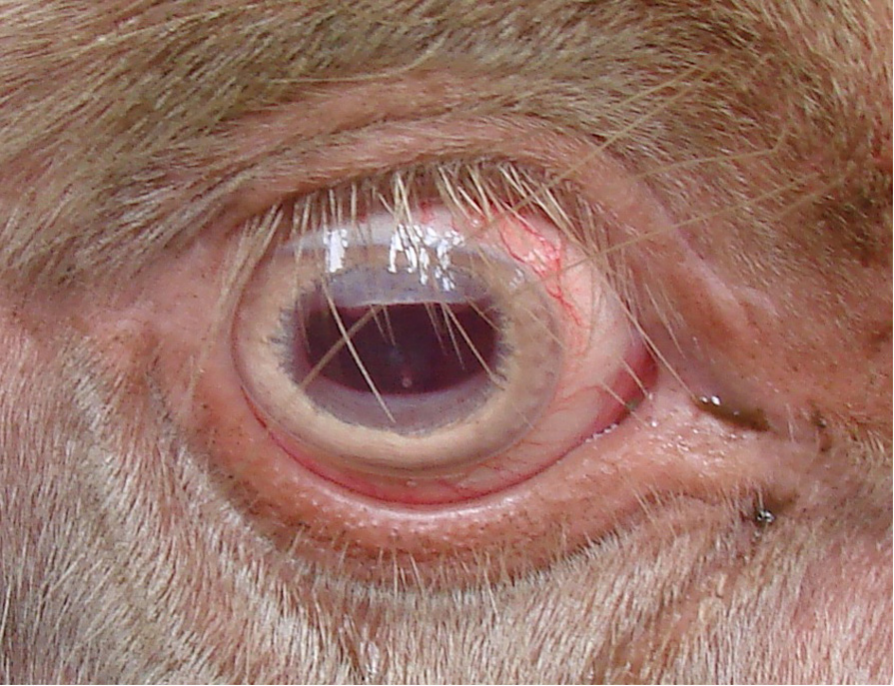
Periocular region in an albino buffalo with non-pigmented eyelashes, conjunctiva, and iris.

In the animals with albinism, the skin and the stratum corneum of the horns (Figure 2) and hooves were white, and the mucosa appeared pink. In the periocular region, an absence of pigmentation was observed in the eyelashes, conjunctiva and iris (Figure 3). The affected buffalo were able to avoid obstacles, but they manifested signs of photophobia by keeping their eyes only partially open, especially when exposed to sunlight.

The TYR mRNA sequence obtained from the wild-type buffalo was 1,958 base pairs (bp) in length (accession number JN_887462), including 39 bp in the 5’-untranslated region (UTR) and 326 bp in the 3’-UTR. The open reading frame of the TYR sequence was 1,593 bp long, and the corresponding peptide sequence consisted of 530 amino acids.

In comparisons of the buffalo TYR coding sequence with the TYR coding sequences from seven other mammalian species, we observed that the length did not vary, except compared to humans (one fewer amino acid) and mice (three additional amino acids). The TYR coding sequence of the wild-type buffalo shared a greater identity with the cattle (98%) and sheep (97%) sequences than with other mammalian TYR sequences deposited in GenBank^TM^.

The TYR gene sequence obtained for the OCA buffalo was deposited in GenBank^TM^ (JN_887463). The sequence was then analyzed for polymorphisms between the OCA and wild-type buffalo. Comparison of the TYR coding regions in the OCA buffalo revealed a single-base substitution at nucleotide 1,431 (G to A), which causes the conversion of a tryptophan (TGG_477_) into a stop codon (TGA_477_). This premature stop codon produces a truncated TYR protein with 53 fewer amino acids than the wild-type TYR protein.

**Figure 4:**
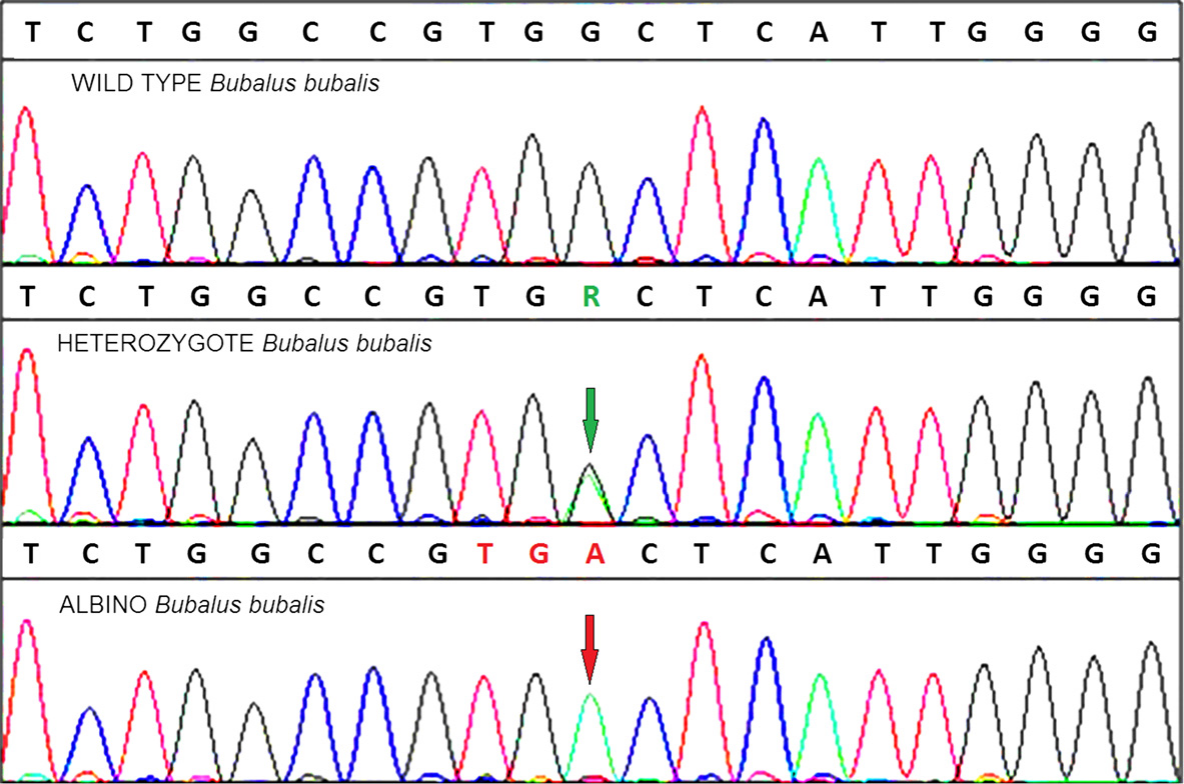
**A partial chromatogram obtained from the assembly of tyrosinase sequences from wild-type, heterozygote and albino buffalo.** The normal G at the 11th nucleotide position shown in the picture was observed in the wild-type TYR sequence. A double peak (R: G/A) was observed in the heterozygote sequence (green arrow), and a point mutation (G to A) forming a premature stop codon (TGA) was observed in the albino sequence (red arrow).

The SNP sequence responsible for OCA in the buffalo was confirmed by PCR using the primers ASBTYR-F3 and ASBTYR-R3. The wild-type, heterozygote and albino sequences obtained in this study were assembled, and the resulting chromatogram is shown in Figure 4.

## Discussion

Albinism was diagnosed in 16 buffalo over a 12-year period. The affected animals exhibited the characteristic phenotype of the disease: a white coat, non-pigmented skin and eyes, and pink mucosa [6,7]. In a previous experiment evaluating another recessive trait [21], some of the sires in the studied herd, including VI-50, were mated several times with their daughters and nieces. This contributed to the increased frequency of albinism among the herd (16/970) compared with the expected frequency if mating were randomly distributed (3/970), considering the expected allelic frequency. This breeding program also contributed to the increase of consanguinity in the herd. The results from the segregation analysis suggest that the recessive allele could have been introduced by the two imported sires. Consanguinity also contributed to other hereditary diseases observed in this herd, including hydranencephaly and cerebellar hypoplasia [21], acantholytic mechanobullous dermatosis [22], and arthrogryposis [23].

Decreased pigmentation in the iris and the retina leads to a diminished ability to absorb light. Light reflects off of normal blood vessels in the back of the eye through the pale iris, resulting in the red eye color found in the studied buffalo and in other species with albinism [6,24]. The photophobia observed in the albino buffalo is the result of insufficient or absent iris pigmentation, which causes increased light-sensitivity and discomfort in the animals when exposed to bright light [24].

Molecular abnormalities in the TYR gene that are responsible for OCA have been described in American mink [3], rabbits [4], chickens [5], cattle [6], whales [8], mice [25], rats [26], cats [7,27,28], and ferrets [29]. In addition, approximately 200 TYR mutations have been described in humans (http://albinismdb.med.umn.edu/) [30]. Therefore, TYR was the first candidate gene to be tested in OCA buffalo, and the results of this study confirmed that TYR was also affected in these animals. However, the mutation reported in this study differed from all of the mutations described in other species.

A frameshift mutation generating a premature stop codon (TGA_491_) resulting in a truncated TYR protein that is shortened by 21 amino acids has been reported in humans [31]. This mutation is found in the putative transmembrane region in exon 5 of the TYR gene (between nucleotides 1,420 and 1,500) and results in the elimination of the carboxy-terminal portion. In buffalo, the detected mutation also occurs in the putative transmembrane region in exon 5 of the TYR gene (nucleotide 1,431), generating a premature stop codon, and as in humans, the carboxy-terminal portion is also eliminated. This region contains a short amino acid sequence (serine-histidine-leucine) that acts as a targeting signal for the transport of several peroxisomal enzymes into peroxisomes [31,32].

The premature stop codon eliminates the targeting signal in humans [31] and most likely has the same effect in buffalo, thereby compromising the insertion and transport of the protein within the melanosome (considered to be modified peroxisomes) membrane and inactivating the tyrosinase enzyme. Nonsense-mediated mRNA decay (NMD) is a quality control mechanism that detects and degrades mRNAs containing premature termination codons. If translated, these mRNAs can produce truncated proteins with dominant-negative or deleterious gain-of-function activities [33,34]. This mechanism has implications for human diseases [35,36] and has also been associated with a mutation resulting in a frameshift and a premature stop codon (exon 1) in the chicken sex-linked allele that causes imperfect albinism [37]. In the albino buffalo, the results of the RNA sequence evaluation indicate that this mechanism is most likely ineffective, as mRNAs with nonsense mutations in the final exon escape NMD because there is no intron downstream of the premature stop codon [34,38-40].

## Conclusion

In buffalo, albinism is an autosomal recessive disease. The detected premature stop codon (G1431A) produces an inactive protein, which is responsible for the OCA buffalo phenotype.

## Competing interests

The authors declare that there are no conflicts of interest, finance or relationships with other people or organizations that could inappropriately influence their work.

## Authors’ contributions

ASB, FRC and ALS designed the study. MCFD and ALS performed the clinical examination and studied the albino buffalo herd. GMX and JPOF performed the molecular study. HNO performed the segregation analyses. All authors reviewed the literature, prepared the manuscript, and approved the final manuscript.

## Authors’ information

MCFD is from the Brazilian Agricultural Research Corporation – Embrapa, Pelotas, Brazil. GXM and FRC are from the Veterinary Hospital of the Federal University of Campina Grande, Patos, Brazil. JPOF and ASB are from the Laboratory of Molecular Biology of the Department of Veterinary Clinical Science / College of Veterinary Medicine and Animal Science, Univ. Estadual Paulista (Unesp), Botucatu, Brazil. HNO is from the Departamento de Zootecnia / Faculdade de Ciências Agrárias e Veterinárias, Univ Estadual Paulista (Unesp), Jaboticabal, Brazil. ALS is from the Veterinary Diagnostic Laboratory of the Federal University of Pelotas, Pelotas, Brazil.
